# Association between triglyceride-glucose index and carotid atherosclerosis detected by ultrasonography

**DOI:** 10.1186/s12933-022-01570-0

**Published:** 2022-07-21

**Authors:** Wenzhen Li, Dajie Chen, Yueqing Tao, Zuxun Lu, Dongming Wang

**Affiliations:** 1 Department of Social Medicine and Health Management, School of Public Health, Tongji Medical College, Huazhong University of Science and Technology, Wuhan, 430030 Hubei People’s Republic of China; 2 Department of Occupational & Environmental Health, School of Public Health, Tongji Medical College, Huazhong University of Science and Technology, No. 13 Hangkong Road, Wuhan, 430030 Hubei China; 3 Key Laboratory of Environment and Health, Ministry of Education & Ministry of Environmental Protection, State Key Laboratory of Environmental Health (Incubating), School of Public Health, Tongji Medical College, Huazhong University of Science and Technology, Wuhan, 430030 Hubei China

**Keywords:** TyG index, Carotid atherosclerosis, Carotid plaques, Carotid intima-media thickness, Carotid stenosis

## Abstract

**Background:**

Several previous studies have indicated that the triglyceride-glucose index (TyG) index is associated with carotid atherosclerosis (CA); however, the evidence of the association is limited and inconsistent, which may result from small sample sizes or differences in study populations. Therefore, we examined the relation between the TyG index and CA in a large general population of Chinese middle-aged and elderly population.

**Methods:**

A total of 59,123 middle-aged and elderly participants were enrolled. The TyG index was calculated as ln[fasting triglycerides (mg/dL)×fasting glucose (mg/dL)/2]. Logistic regression models were used to analyze the relationship between the TyG index as continuous variables and quartiles and CA. The relationships between the TyG index and CA according to sex, age groups, blood pressure groups and body mass index groups were also assessed.

**Results:**

The multivariate logistic regression analysis showed that the TyG index was significantly associated with the prevalence of CA (OR: 1.48; 95% CI 1.39–1.56), carotid intima-media thickness (CMT) (1.55; 1.45–1.67), plaques (1.38; 1.30–1.47) and stenosis severity (> 50%) (1.33; 1.14–1.56). Compared with the quartile 1, quartile 4 was significantly associated with a higher prevalence of CA (1.59; 1.45–1.75), CMT (1.93; 1.82–2.18), plaques (1.36; 1.22–1.51) and stenosis severity (> 50%) (1.56; 1.20–2.04). Subgroup analyses showed significant associations between the continuous TyG index and the prevalence of CA, CMT, plaques and stenosis severity (> 50%) according to sex, with a higher prevalence of CA, CMT, and plaques among males, while a higher prevalence of stenosis severity in females (> 50%). For participants aged < 60 years old and with hypertension, the relationship between the TyG index and stenosis severity (> 50%) was not observed (1.47; 0.97–2.22 and 1.13; 0.91–1.41). For body mass index (BMI), the association was just observed among overweight participants (1.48; 1.17–1.86). In addition, similar results were also observed when the TyG index was used as a categorical variable.

**Conclusions:**

There is a positive association between the TyG index and CA. The association is higher in males and middle-aged individuals than those in females and elderly individuals. Besides, the relationship is stronger among individuals with normal blood pressure and underweight subjects.

## Introduction

Cardiovascular diseases (CVDs) comprised 31% of all global deaths, with estimated 17.9 million deaths worldwide in 2015, and it was estimated that by 2030, approximately 23.6 million people will die of CVDs annually [1]. Carotid atherosclerosis (CA) including plaque, stenosis and carotid intima-media thickness (CMT), is the most important process of CVDs and could be assessed non-invasively with ultrasonography [2], and it has been considered as a key indicator of CVD risk. The still increasing burden of CVDs indicates an urgent need for early detection of CA and among apparently healthy populations, as well as for determining possible biomarkers and implementation of preventive measures.

The triglyceride-glucose (TyG) index, calculated as ln (fasting triglycerides (mg/dl) × fasting blood glucose (mg/dl)/2), has become a valuable biomarker for insulin resistance [3]. Previous studies have shown that the TyG index was associated with cardiovascular events [4]. For instance, the TyG index was associated with the prevalence of coronary artery disease (CAD) [5] and an increased risk of ischemic stroke [6], hypertension [7], and arterial stiffness [8]. However, only a few studies have focused on the relationship between the TyG index and CA among the general population, and the results have been inconsistent. C Irace’s study [9] published in 2013 showed that the TyG index was associated with CA with 1,432 subjects; however, detailed analyses, such as different CA types or subgroups were not conducted. Zhu Li’s study [10] was conducted in 10,535 patients with coronary heart disease, who reported that the TyG index was related to carotid artery plaques; however, CMT was not assessed in the study. Another study [11] was conducted among 2,830 people aged 65 years or older and did not observe a relationship between the TyG index and CA. In addition, a cut-off of CMT > 0.9 mm, not 1.0 mm, was used in the study, which may overestimate the prevalence of CA.

Considering the inconsistent results and small sample size, we aimed to examine the association between the TyG index and carotid atherosclerosis, including carotid artery plaque, CMT, and stenosis severity among 59,123 general middle-aged and elderly populations, as well as to explore and clarify possible characteristic populations by conducting subgroup analyses, which will contribute to the development and implementation of preventive measures.

## Methods

### Study population

Participants were from the Stroke Screening and Prevention Project in Hubei Province, China from 2017–2020. The cluster sampling method was used in the project. Five cities with 9 communities were selected in proportion to the local population size and the numbers of communities, and all residents aged ≥ 40 years were surveyed during the primary screening. Community physicians collected information on participants’ sociodemographic characteristics during in-person interviews firstly, and then, participants were invited for further physical examination, including blood pressure measurements, laboratory tests, electrocardiography, and carotid ultrasound. A total of 61,638 participants were included in the survey from 2017–2020, and 982 were excluded due to missing sociodemographic information. Among 60,656 subjects, 59,123 participants underwent laboratory tests and carotid ultrasound with full data were included in the present analyses. Carotid ultrasonography examination was performed by qualified ultrasound technologists using one of the ultrasound systems (Logiq 9 [GE Healthcare], iU22 [Philips Healthcare], S2000 [Siemens Medical Solutions]). Linear array probes with a transmission frequency of 6 to 10 MHz were used, and all procedures were conducted according to Chinese stroke vascular ultrasound examination guidelines.

### Definition of variables

Demographic characteristics including age, sex, educational level, behavioral risk factors such as smoking, drinking, physical activity and history of diseases including cerebrovascular disease, heart diseases, hypertension, dyslipidemia and diabetes were investigated by trained staff with face-to-face interviews in primary healthcare institutions. Smoking was defined as smoking at least one cigarette per day for more than half a year. Drinking was defined as regular heavy drinking (≥ 3 times/week). Physical activity was defined as regular physical exercise > 3 times/week for at least 30 min per session.

Physical examinations included the measurement of body weight, height, and blood pressure. Body mass index (BMI) was calculated as body weight (kg) divided by the square of height (kg/m^2^). Blood pressure (BP) was calculated using the average of 3 measurements at 1-minute intervals after 5 min of rest. Normal BP was defined as systolic blood pressure (SBP) ≤ 130 mm Hg and diastolic blood pressure (DBP) ≤ 85 mm Hg, and hypertension was defined as systolic blood pressure ≥ 140 mm Hg, and/or diastolic blood pressure ≥ 90 mm Hg, and/or self-reported hypertension diagnosed by a physician, and/or the use of antihypertensive medications. Blood samples were collected to test fasting plasma glucose (FPG), triglyceride (TG), total cholesterol (TC), low-density lipoprotein cholesterol (LDL-C), and high-density lipoprotein cholesterol (HDL-C).

Participants with increased CMT or plaques were defined as having CA. Increased CMT was defined as CMT ≥ 1.0 mm in either the left or right carotid artery. Plaque was defined as CMT ≥ 1.5 mm or focal narrowing of the vessel wall of > 50% relative to adjacent segments. Stenosis severity was classified into the following categories: normal (no stenosis), < 50%, 50–69%, 70–99%, and occlusion. In the present study, stenosis was defined as ≥ 50% stenosis. When the bilateral carotid arteries were measured, we used the most severe stenosis to grade the severity.

### Statistical analysis

Continuous variables were shown as the mean [standard deviation (SD)] and qualitative variables were presented as numbers with percentages in parentheses. The characteristics of the participants in the different groups were compared by the chi-squared test. The quantitative parameters of males and females were compared by Student’s t test. The participants were divided into four groups according to the quartile level of the TyG index. The odds ratio (OR) and 95% confidence interval (CI) was used to evaluate the association of the TyG index and CA. Three logistic models were performed in the present study: (a) crude; (b) adjusted for sex, age, education, smoking, drinking, physical activity; and (c) adjusted for sex, age, education, smoking, drinking, physical activity, BMI, SBP, DBP, TC, LDL-C, HDL-C, history of diseases including cerebrovascular disease, heart diseases, hypertension, dyslipidemia and diabetes. Furthermore, stratified analyses were conducted between different types of BP, sex and BMI in the subgroup participants. Meanwhile, linear trend tests were conducted by including the median value as a continuous variable in the models. All analyses were performed using SAS software version 9.4 (SAS Institute, Inc., Cary, North Carolina, USA). The statistical tests were two sided, and significance was *P* < 0.05.

## Results

### Characteristics of the study participants

Table 1 showed the basic characteristics of the 59,123 participants included in our study. The mean age of these participants was 60.03 (10.75) years old, and 26,197 subjects were males. Among them, 1,678 (2.84%), 19,97 (3.38%), 8,471 (14.33%) and 8,422 (14.42%) had history of cerebrovascular disease, heart disease, dyslipidemia and diabetes mellitus, respectively. A total of 8,862 participants consumed alcohol and 7,590 smoking, and 38,345 subjects had physical activity. We observed significant differences in SBP, DBP, TG, TC, LDL-C, HDL-C and CA, CMT, plagues between males and females (all *P* < 0.001), while no significant difference was observed in FPG (*P* = 0.468).

**Table 1:** Characteristics of participants in the study

	Overall (59,123)	Male (26,197)	Female (32,926)	*p*-value
Age (years)	60.03 (10.75)	60.11 (10.92)	59.96 (10.61)	0.102
Education				< 0.001
Primary school and below	15,747 (26.63)	6077 (23.20)	9670 (29.37)	
Junior middle school	23,238 (39.30)	10,267 (39.19)	12,971 (39.39)	
Senior high school	13,624 (23.04)	6250 (23.86)	7374 (22.40)	
College and above	6514 (11.02)	3603 (13.75)	2911 (8.84)	
Smoking				< 0.001
No	51,533 (87.16)	19,048 (72.71)	32,485 (98.66)	
Yes	7590 (12.84)	7149 (27.29)	441 (1.34)	
Drinking				< 0.001
No	50,261 (85.01)	18,854 (73.36)	30,097 (93.72)	
Yes	8862 (14.99)	6845 (26.64)	2017 (6.28)	
Physical activity				< 0.001
No	20,778 (35.14)	9625 (36.74)	11,153 (33.87)	
Yes	38,345 (64.86)	16,572 (63.26)	21,773 (66.13)	
History of diseases				
Cerebrovascular disease				0.105
No	57,445 (97.16)	25,421 (97.04)	32,024 (97.26)	
Yes	1678 (2.84)	776 (2.96)	902 (2.74)	
Heart disease				0.388
No	57,126 (96.62)	25,331 (96.69)	31,795 (96.57)	
Yes	1997 (3.38)	866 (3.31)	1131 (3.43)	
Dyslipidemia				0.147
No	50,652 (85.67)	22,505 (85.91)	28,147 (85.49)	
Yes	8471 (14.33)	3692 (14.09)	4779 (14.51)	
Diabetes mellitus				0.565
No	50,701 (85.76)	22,441 (85.66)	28,260 (85.83)	
Yes	8422 (14.42)	3756 (14.34)	4666 (14.17)	
BMI	23.98 (3.28)	24.18 (3.11)	23.82 (3.40)	< 0.001
SBP	130.15 (16.73)	130.75 (17.46)	129.75 (17.46)	< 0.001
DBP	79.58 (10.24)	80.82 (10.19)	78.60 (10.17)	< 0.001
FPG	5.56 (1.75)	5.57 (1.78)	5.56 (1.72)	0.468
TG	1.66 (0.98)	1.67 (1.02)	1.64 (0.94)	< 0.001
TC	4.72 (1.33)	4.56 (1.32)	4.85 (1.33)	< 0.001
LDL-C	2.64 (0.91)	2.54 (0.89)	2.71 (0.91)	< 0.001
HDL-C	1.44 (0.90)	1.38 (0.54)	1.48 (1.10)	< 0.001
CA				< 0.001
No	53,290 (90.13)	23,115 (88.24)	30,175 (91.64)	
Yes	5833 (9.87)	3082 (11.76)	2751 (8.36)	
CMT				< 0.001
No	55,725 (94.25)	24,289 (92.72)	31,436 (95.47)	
Yes	3398 (5.75)	1908 (7.28)	1490 (4.53)	
Plaques				< 0.001
0	54,813 (92.71)	23,918 (91.30)	30,895 (93.83)	
1	1948 (3.29)	958 (3.66)	990 (3.01)	
≥ 2	2362 (4.00)	1321 (5.04)	1041 (3.16)	
Stenosis (≥ 50%)				< 0.001
No	58,543 (99.02)	25,862 (98.72)	32,681 (99.26)	
Yes	580 (0.98)	335 (1.28)	245 (0.74)	

*BMI* body mass index; *SBP* systolic blood pressure; *DBP* diastolic blood pressure; *FPG* fasting plasma glucose; *TG* triglyceride; *TC* total cholesterol; *LDL-C* low-density lipoprotein cholesterol; *HDL-C* high-density lipoprotein cholesterol; *CA* carotid atherosclerosis; *CMT* carotid intima–media thickness

### Association between the TyG index and the prevalence of CA

The results of multivariate logistic regression analysis were shown in Table 2. When the TyG index was used as a continuous variable, it was significantly associated with the prevalence of CA (OR: 1.48; 95% CI 1.39–1.56), CMT (1.55; 1.45–1.67), plaques (1.38; 1.30–1.47) and stenosis severity (> 50%) (1.33; 1.14–1.56). Compared with the Q1, Q4 was significantly associated with a higher prevalence of CA (1.59; 1.45–1.75, *P*_*for trend*_ <0.001), CMT (1.93; 1.82–2.18, *P*_*for trend*_ <0.001), plaques (1.36; 1.22–1.51, *P*_*for trend*_ <0.001) and stenosis severity (> 50%) (1.56; 1.20–2.04, *P*_*for trend*_ <0.001).

**Table 2:** Odds ratios and 95% CIs for the association of the TyG index with CA, CMT, plaques and stenosis

	Continuous	Quartiles of the TyG index				*P* for trend
		Q1	Q2	Q3	Q4	
CA						
Model 1	1.94 (1.86–2.04)	Ref	1.08 (0.99–1.18)	1.49 (1.37–1.62)	2.42 (2.24–2.62)	< 0.001
Model 2	1.95 (1.85–2.05)	Ref	1.11 (1.01–1.22)	1.57 (1.44–1.72)	2.48 (2.28–2.70)	< 0.001
Model 3	1.48 (1.39–1.56)	Ref	0.98 (0.89–1.08)	1.27 (1.15–1.40)	1.59 (1.45–1.75)	< 0.001
CMT (≥ 1.00 mm)						
Model 1	1.98 (1.87–2.10)	Ref	1.23 (1.10–1.39)	1.78 (1.59–1.98)	2.77 (2.49–3.07)	< 0.001
Model 2	1.93 (1.81–2.05)	Ref	1.24 (1.10–1.40)	1.79 (1.60–2.01)	2.71 (2.43–3.02)	< 0.001
Model 3	1.55 (1.45–1.67)	Ref	1.14 (1.01–1.29)	1.63 (1.36–1.73)	1.93 (1.82–2.18)	< 0.001
Plaques						
Model 1	1.84 (1.75–1.94)	Ref	1.01 (0.92–1.12)	1.23 (1.12–1.35)	2.12 (1.94–2.31)	< 0.001
Model 2	1.84 (1.74–1.94)	Ref	1.04 (0.94–1.16)	1.30 (1.18–1.44)	2.17 (1.97–2.38)	< 0.001
Model 3	1.38 (1.30–1.47)	Ref	0.92 (0.82–1.02)	1.03 (0.93–1.15)	1.36 (1.22–1.51)	< 0.001
Stenosis (≥ 50%)						
Model 1	1.74 (1.52–1.99)	Ref	0.91 (0.69–1.20)	1.46 (1.14–1.88)	2.28 (1.81–2.88)	< 0.001
Model 2	1.64 (1.43–1.89)	Ref	0.86 (0.64–1.16)	1.48 (1.14–1.91)	2.11 (1.65–2.68)	< 0.001
Model 3	1.33 (1.14–1.56)	Ref	0.83 (0.62–1.12)	1.34 (1.03–1.76)	1.56 (1.20–2.04)	< 0.001

*CA* carotid atherosclerosis; *CMT* carotid intima–media thickness; *TyG* triglyceride-glucose

Model 1: Unadjusted;

Model 2: Adjusted for sex, age, education, smoke, drink, physical activity;

Model 3: Adjusted for sex, age, education, smoke, drink, physical activity, BMI, SBP, DBP, TC, LDL-C, HDL-C, history of diseases including cerebrovascular disease, heart diseases, hypertension, dyslipidemia and diabetes

### Subgroup analyses for the association between the TyG index and the prevalence of CA

**Fig. 1:**
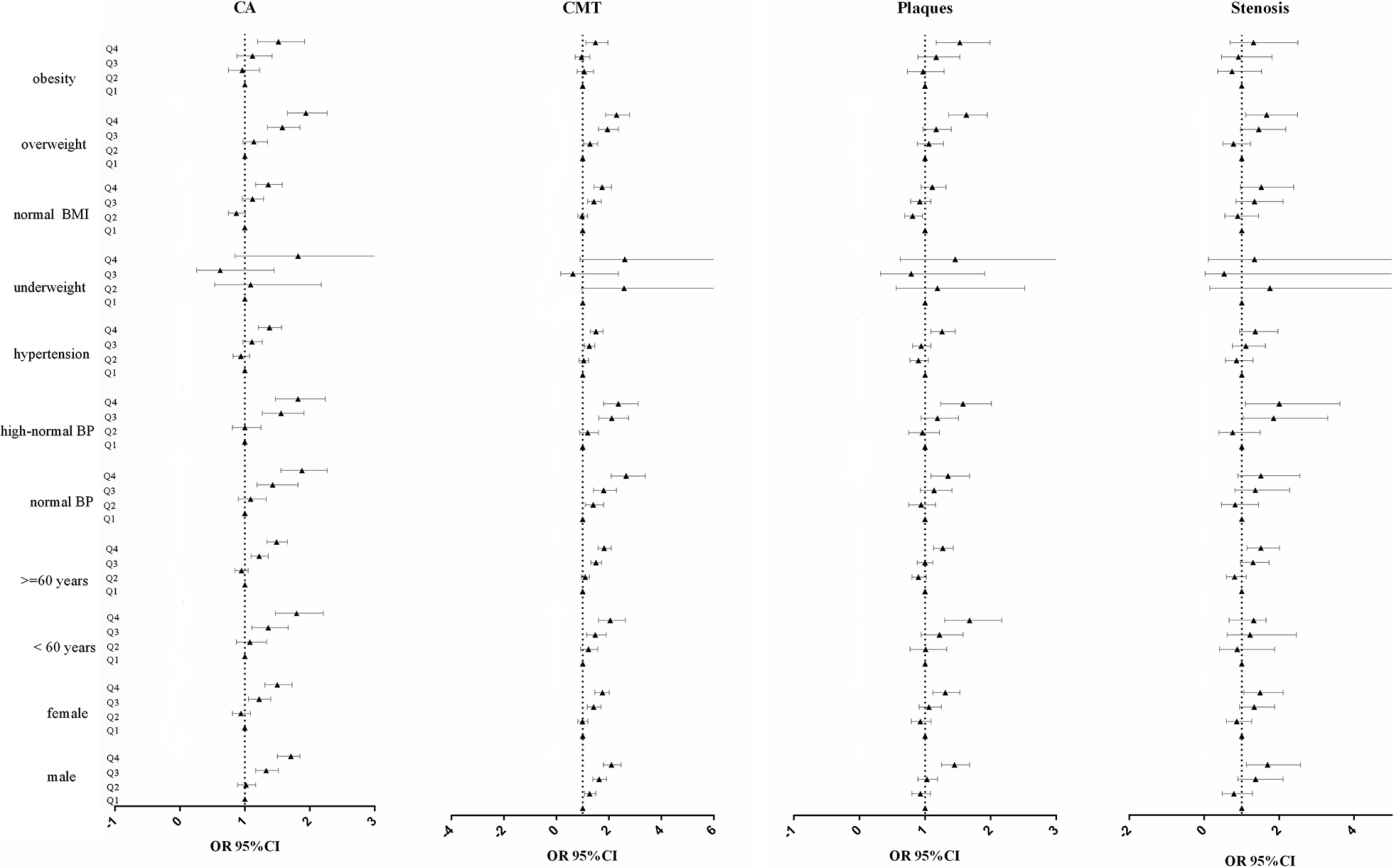
Subgroup analyses for the association of the quartiles of TyG index with CA, CMT, plaques and stenosis. Adjusted for sex, age, education, smoke, drink, physical activity, BMI, SBP, DBP, TC, LDL-C, HDL-C, history of diseases including cerebrovascular disease, heart diseases, hypertension, dyslipidemia and diabetes

Table 3 showed the subgroup analyses for the association between the continuous TyG index and the prevalence of CA, results after multivariate adjustment showed significant associations between the TyG index and the prevalence of CA, CMT, plaques and stenosis severity (> 50%) according to sex, with a higher prevalence of CA, CMT, and plaques among males while a higher risk of stenosis severity in females (> 50%). For participants aged < 60 years old and with hypertension, the relationship between the TyG index and stenosis severity (> 50%) was not observed (1.47; 0.97–2.22 and 1.13; 0.91–1.41). For BMI, the association was just observed among overweight participants (1.48; 1.17–1.86). In addition, similar results were observed when the TyG index was used as a categorical variable (Fig. 1).

**Table 3:** Subgroup analyses for the association of the continuous TyG index with CA, CMT, plaques and stenosis

	OR, 95% CI			
	CA	CMT	Plaques	Stenosis (≥ 50%)
Sex				
Male (N = 26,197)	1.55 (1.43–1.68)	1.60 (1.46–1.76)	1.46 (1.33–1.60)	1.27 (1.04–1.56)
Female (N = 32,926)	1.41 (1.30–1.53)	1.51 (1.36–1.67)	1.32 (1.20–1.45)	1.44 (1.13–1.84)
Age				
< 60 (N = 30,593)	1.63 (1.45–1.84)	1.70 (1.48–1.95)	1.74 (1.50–2.01)	1.47 (0.97–2.22)
≥ 60 (N = 28,530)	1.41 (1.32–1.50)	1.48 (1.37–1.60)	1.29 (1.20–1.39)	1.27 (1.07–1.50)
Blood pressure				
Normal (N = 28,249)	1.67 (1.49–1.88)	1.91 (1.66–2.20)	1.45 (1.27–1.66)	1.38 (1.01–1.90)
High-normal BP (N = 14,571)	1.62 (1.43–1.83)	1.80 (1.54–2.09)	1.48 (1.28–1.71)	1.84 (1.32–2.58)
Hypertension (N = 16,303)	1.33 (1.23–1.44)	1.33 (1.21–1.46)	1.31 (1.20–1.43)	1.13 (0.91–1.41)
BMI				
Underweight (N = 1174)	1.61 (1.01–2.57)	1.86 (1.04–3.35)	1.27 (0.76–2.12)	2.99 (0.60-14.96)
Normal (N = 30,570)	1.36 (1.24–1.50)	1.50 (1.33–1.69)	1.21 (1.09–1.35)	1.25 (0.95–1.66)
Overweight (N = 22,352)	1.60 (1.47–1.75)	1.64 (1.48–1.83)	1.55 (1.40–1.72)	1.48 (1.17–1.86)
Obesity (N = 5027)	1.46 (1.27–1.67)	1.38 (1.18–1.61)	1.47 (1.27–1.71)	1.07 (0.73–1.58)

*CA* carotid atherosclerosis;* CMT* carotid intima–media thickness;* BMI* body mass index;* TyG* triglyceride-glucose

Adjusted for sex, age, education, smoke, drink, physical activity, BMI, SBP, DBP, TC, LDL-C, HDL-C, history of diseases including cerebrovascular disease, heart diseases, hypertension, dyslipidemia and diabetes

## Discussion

The present study determined a significant relationship between the TyG index and CA, CMT, plaques and stenosis among the Chinese middle-aged and elderly population. It was the first large sample size study to demonstrate the association between the TyG index and CA among the general population, and to evaluate the relationship based on sex, age, BP and BMI. As a credible surrogate marker of insulin resistance, the TyG index is simple to calculate without additional costs or non-routine tests [12, 13], which could become a simple but effective tool for risk assessment in daily clinical practice, as well as be used among healthy populations by self-evaluation [14].

To our knowledge, the present study is the largest population to examine the correlation between the TyG index and CA. Our results are consistent with previous studies, which have shown a relationship between the TyG index and carotid artery intima-media thickness in a small Brazilian population [15] and CA among Italy population [9]. However, another study [11] did not find the association between the TyG index and CMT or carotid plaque. In the present study, significant association of the TyG index and plaques was found. Plaque, as an index of atherosclerosis, is easy to be studied non-invasively when it exists in carotid district, previous studies indicated that plaques in carotid district correlates well with atherosclerosis in other districts, carotid plaque may give an overall estimate of the degree of atherosclerosis [16, 17]. Furthermore, previous studies indicated that the TyG index was also independently associated with arterial stiffness [18, 19]. Thus, the management of the TyG index may be an effective evidence-based medical intervention that reduce atherosclerosis progresses and CVD risk [20, 21], especially for subclinical atherosclerosis population.

Subgroup analyses found that the TyG index was also significantly associated with the progression of stenosis (> 50%) in people aged ≥ 60-years but not among individuals aged < 60 years. A higher OR of CA including CMT and plaques was observed in males and people aged < 60 years, which indicated that more attention should be paid to them. Our study was inconsistent with a previous study, which showed a higher prevalence in female than in male and elderly (> 60 years old) patients with coronary heart disease [10], which may result from the different populations, and our study enrolled the general population. Besides, it is interesting that the lowest OR between the TyG index and CA was found in the hypertension population, which was inconsistent with a previous study, which indicated that the TyG index was significantly associated with the progression of arterial stiffness in hypertensive people but not prehypertensive individuals [8]. One possible explanation is that arterial stiffness is different from CA, CA reflects structural changes in the artery wall [22], and arterial stiffness was assessed by pulse wave velocity. Another explanation may be that elevated blood pressure may force individuals to make some changes, such as control weight, healthy diets; however, more studies should be conducted to explore the reasons. Furthermore, our study reveals a stronger relationship between the TyG index and CA and CMT among the underweight population, not in obese people, which implies that underweight individuals should not be ignored by themselves and clinical practitioners.

Some potential mechanisms may contribute to understanding the relationship between the TyG index and CA. The TyG index is associated with inflammation, and the TyG index has been suggested as a convenient insulin resistance marker [14]. Insulin resistance could induce oxidative stress, inflammation and metabolic changes, leading to damage to the vascular endothelium [23]. Therefore, the TyG index was associated with CA, and a high TyG index was associated with a higher prevalence of CA. However, further studies are needed to explore and determine the mechanisms.

Several limitations need to be interpreted. First, this is a cross-sectional study, and we could not infer the causality of the results. Second, the study included only Chinese middle-aged and elderly participants, so we should be cautious in extrapolating the present findings to other populations. Third, the potential mechanism of the relationship between the TyG index and CA requires further prospective large-scale research. Finally, some dietary factors that might impact the results were not included in our investigation. Last but not least, gender difference is not highlighted in the present study, which should be explored in the further study.

## Conclusions

There is a positive association between the TyG index and CA. The association is higher in males and middle-aged individuals than those in females and elderly individuals. Besides, the relationship is stronger among individuals with normal blood pressure and underweight subjects.

## Data Availability

The datasets used and/or analyzed during the current study are available from the corresponding author on reasonable request.

## Abbreviations

CVD: Cardiovascular diseases
CA: Carotid atherosclerosis
CMT: Carotid intima-media thickness
TyG: Triglyceride glucose index
CAD: Coronary artery disease
BMI: Body mass index
BP: Blood pressure
SBP: Systolic blood pressure
DBP: Diastolic blood pressure
FPG: Fasting plasma glucose
TG: Triglyceride
TC: Total cholesterol
LDL-C: Low-density lipoprotein cholesterol
HDL-C: High-density lipoprotein cholesterol
SD: Standard deviation
OR: Odds ratio
CI: Confidence interval
